# Lyme Borreliosis–Biology, Epidemiology and Control

**DOI:** 10.3201/eid1003.030686

**Published:** 2004-03

**Authors:** Ira Schwartz

**Affiliations:** * New York Medical College, Valhalla, New York, USA

Lyme borreliosis is the most common arthropod-borne disease in the United States and Europe, and cases are reported from nearly all regions of the Northern Hemisphere. As such, it is a particularly useful example of a zoonotic disease that should be considered not only from the perspective of the human patient but also from that of the vectors and reservoir hosts. This approach is the one adopted by the editors of Lyme Borreliosis–Biology, Epidemiology and Control. The first five chapters constitute a general review covering characteristics of the disease, molecular biology of the etiologic agent *Borrelia burgdorferi* sensu lato, and vector and host biology. Some of these chapters are not reviews in the truest sense. For example, chapter 2 is a primer on ecologic research; it provides important definitions and categorization of hosts and vectors. Likewise, a substantial portion of the chapter on *B. burgdorferi* in the vertebrate host is devoted to the role of complement in host specificity. Still, these chapters provide a good general description of the topics covered.

Chapters 6–9 provide more specific detail on the unique aspects of ecology in Europe, Russia, Japan, and North America, respectively. Although these chapters do not follow the same format, all cover essentially identical topics, including description of the diversity of spirochete species, vectors, hosts, landscape ecology, and risk factors. The chapter on the ecology of *B. burgdorferi* in Russia is particularly worthwhile because many of the studies described have only been published to date in Russian-language journals. This information is now available to a broader audience.

The final three chapters deal with epidemiology, prevention, and control. The chapter by Dennis and Hayes on epidemiology is outstanding and provides an excellent consideration of several controversial topics, including seronegative Lyme disease, persistent infection, chronic refractory Lyme disease, and problems of both underdiagnosis (in Lyme disease–endemic areas) and overdiagnosis. Hayes and Schriefer provide a comprehensive description of vaccine development. Unfortunately, the only commercially available vaccine, Lymerix, has been discontinued by the manufacturer, and little progress is reported on development of alternative vaccines. This leaves personal protective measures (e.g., avoiding tick-infested terrain, using appropriate clothing, checking for attached ticks), single-dose antimicrobial prophylaxis following selected tick bites, and tick control as the primary means of prevention. The last topic is covered in the book’s final chapter.

The reader interested in details of the clinical aspects of Lyme disease will not find them here. Rather, the book focuses on vector and host wildlife ecology and epidemiology, as the title implies. There is an obvious effort to cover Lyme borreliosis from a global perspective rather than from a distinctly American one. This approach is one of the book’s strengths, although it does lead to some bias in the selected references in certain chapters. A comprehensive index is provided, and topics covered in multiple chapters are cross-referenced well, providing an integrated feel. The book will be quite useful for ecologists, epidemiologists, and public health workers, especially those interested in vector-borne infectious diseases. The book will be less informative for those interested in detailed descriptions of the molecular pathogenesis and clinical aspects of Lyme borreliosis.

